# Effects of treadmill running with different intensity on rat subchondral bone

**DOI:** 10.1038/s41598-017-02126-z

**Published:** 2017-05-16

**Authors:** Zhe Li, Sheng-Yao Liu, Lei Xu, Shao-Yong Xu, Guo-Xin Ni

**Affiliations:** 1 Department of Orthopaedics and Traumatology, Zhengzhou Orthopaedics Hospital, Zhengzhou, China; 2 Department of Orthopaedics and Traumatology, Nanfang Hospital, Southern Medical University, Nanfang, China; 3 Department of Rehabilitation Medicine, First Affiliated Hospital, Fujian Medical University, Fujian, China

## Abstract

Subchondral bone (SB) is recognized as a key factor in normal joint protection, not only does it provide a shock absorbing and supportive function for the cartilage, but it may also be important for cartilage metabolism. Mechanical loading is considered to be a critical regulator of skeletal homeostasis, including bone and cartilage. It is suggested that both cartilage and bone may respond to mechanical loading in an intensity-dependent manner. In this report, we have discovered that the subchondral plate became thicker with higher bone mineral density (BMD) and lower porosity, while trabecular bone became more plate-like and denser with higher BMD in high-intensity running (HIR) group. Further, HIR led to highly remodeled, less mineralized, and stiffer subchondral plate and trabecular bone. On the contrary, low-intensity running and moderate-intensity running failed to result in considerable changes in microstructure, composition and hardness. Our findings suggested that running affects SB in an intensity-dependent manner. In addition, HIR may induce change in organization and composition of SB, and consequently alter its mechanical properties. HIR-induced “brittle and stiff” SB may adversely affect the overlying articular cartilage.

## Introduction

Subchondral bone (SB) is the zone of epiphyseal bone just beneath the articular cartilage, and includes the SB plate and the underlying trabecular bone. SB is recognized as a key factor in normal joint protection, not only it provides a shock absorbing and supportive functions for the cartilage, but it may also be important for cartilage metabolism^1^. In fact, the conditions of articular cartilage and its supporting SB are tightly coupled and should be viewed as an authentic functional unit^1, 2^. Recently, there has been an increasing interest and awareness of the importance of the SB for its role in the pathogenic processes^1–3^, as well as the necessity to carefully consider this structure in the treatment of osteoarthritis (OA)^4, 5^.

Mechanical loading is considered to be a critical regulator of skeletal homeostasis, including bone and cartilage. It is suggested that both cartilage and bone may respond to mechanical loading in an intensity-dependent manner^6^. In a rat model, we previously demonstrated that treadmill running with low-to-medium intensity maintains cartilage homeostasis, whereas, high-intensity running may cause cartilage degradation^7^. We therefore hypothesize that the response of SB may differ under different running-induce loadings, and would like to test this hypothesis using the same animal model in the present study.

The objectives of this research are two-fold. One is to investigate the effect of running with different intensities on SB. Although the effect of running on bone has been documented extensively^8–11^, its effect on SB is largely unknown. Intensity may be an important influencing factor to the role of running in bone due to inconsistent findings from the above cited studies^8–11^. Specifically, Murray *et al*. reported that equine subchondral bone thickness, hardness and remodelling are influenced by exercise intensity^12^. However, little is known about changes in microarchitecture and composition of SB, which will be examined using micro-CT and Raman spectroscopy in the present study, respectively.

Due to the important role of SB in OA pathogenesis, changes in SB have been investigated in a number of OA animal models^13, 14^. Since OA is thought to be a multifactorial disease, it is necessary to use multiple models to best understand its disease progression. As above mentioned, high-intensity running would lead to OA-like changes. However, inconsistent changes of SB have been reported during OA initiation and progression, which are largely due to different types of model used^13–15^. Therefore, the second objective of this study is to understand changes in SB in this running-induced OA animal model.

## Results

### Results from Micro-CT

#### Subchondral plate

**Figure 1:**
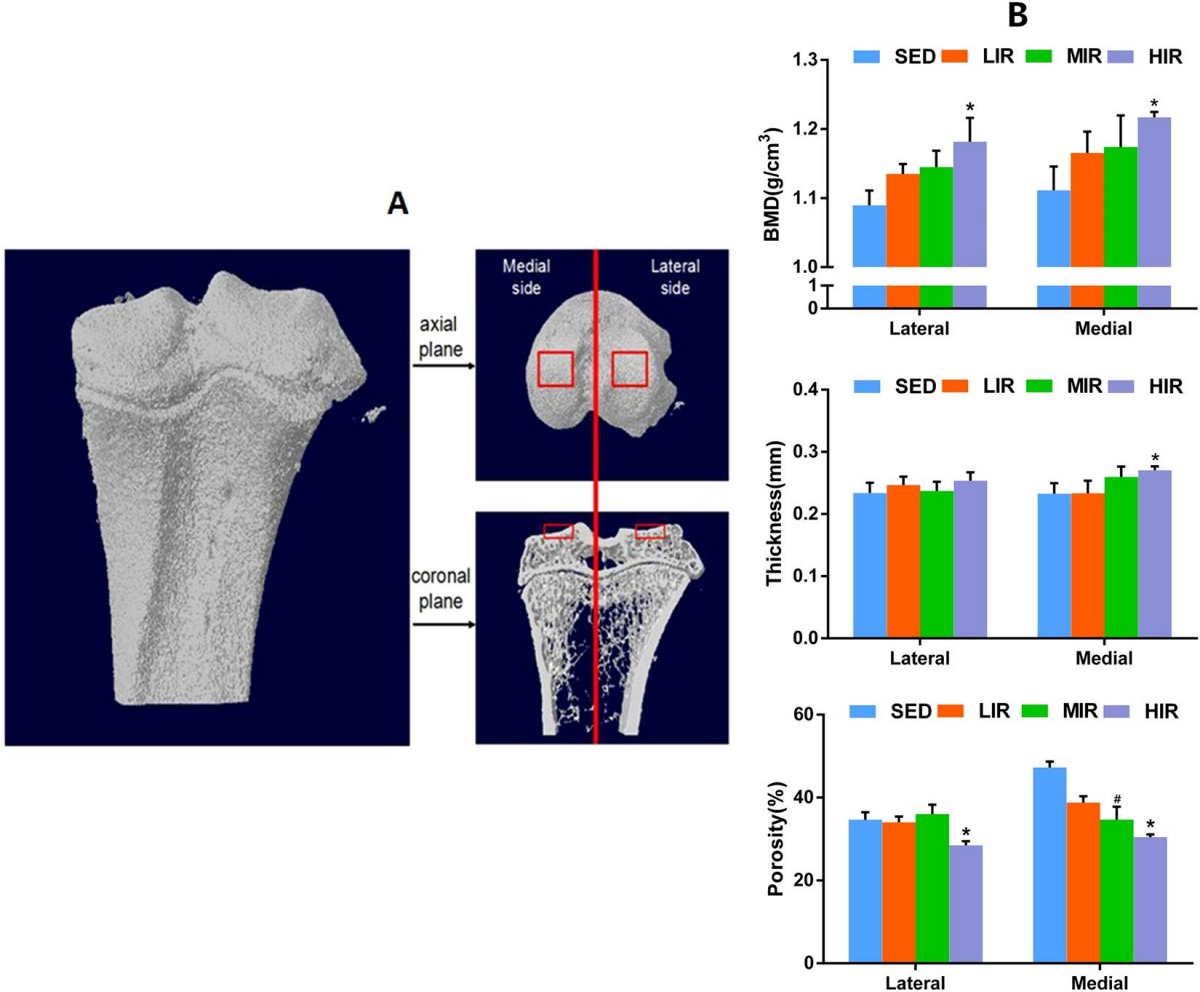
Micro-CT analysis of tibia subchondral plate. (**A**) Demographic images of top and coronal views of ROI selection of medial and lateral tibia subchondral plate. (**B**) The results of BMD, thickness and porosity of medial and lateral subchondral plate in four groups. *p < 0.05 compared to SED group at corresponding side. ^#^p < 0.05 compared to HLR group at corresponding side.

Figure 1A illustrates the 3-D reconstruction image of the proximal end of left tibia, and the demographic images of top and coronal views of region of interest (ROI) selection of medial and lateral tibia subchondral plate. Figure 1B shows the results of bone mineral density (BMD), thickness and porosity of medial and lateral subchondral plate in four groups. In comparison with sedentary control (SED) group, a significantly higher BMD was observed in high-intensity running (HIR) group on both lateral (1.181 ± 0.034 g/cm^3^ for HIR group and 1.089 ± 0.021 g/cm^3^ for SED group, p = 0.030), and medial sides (1.217 ± 0.007 g/cm^3^ for HIR group and 1.111 ± 0.034 g/cm^3^ for SED group, p = 0.050), respectively. On the medial side, subchondral plate was significantly thicker in HIR group (0.271 ± 0.006 mm) than that in SED group (0.232 ± 0.016 mm) (p = 0.037). On the lateral side, thicker subchondral plate was also found in group HIR (0.253 ± 0.033 mm) compared with group SED (0.233 ± 0.041 mm) (p = 0.067), but without statistical difference. A significantly lower porosity of subchondral plate was defined in HIR group than SED group on both the lateral side (28.47 ± 0.97% vs. 34.69 ± 1.76%, p = 0.047) and medial side (30.48 ± 0.64% vs. 47.22 ± 1.46%, p = 0.001). Considerably lower porosity was also found in moderate-intensity running (MIR) group than SED group on the medial side (34.68 ± 3.11% vs. 47.22 ± 3.63%, p = 0.005). Nevertheless, compared with group SED, there was no statistical difference in groups low-intensity running (LIR) and MIR, in either BMD, or thickness, respectively.

#### Subchondral trabecular bone

**Figure 2:**
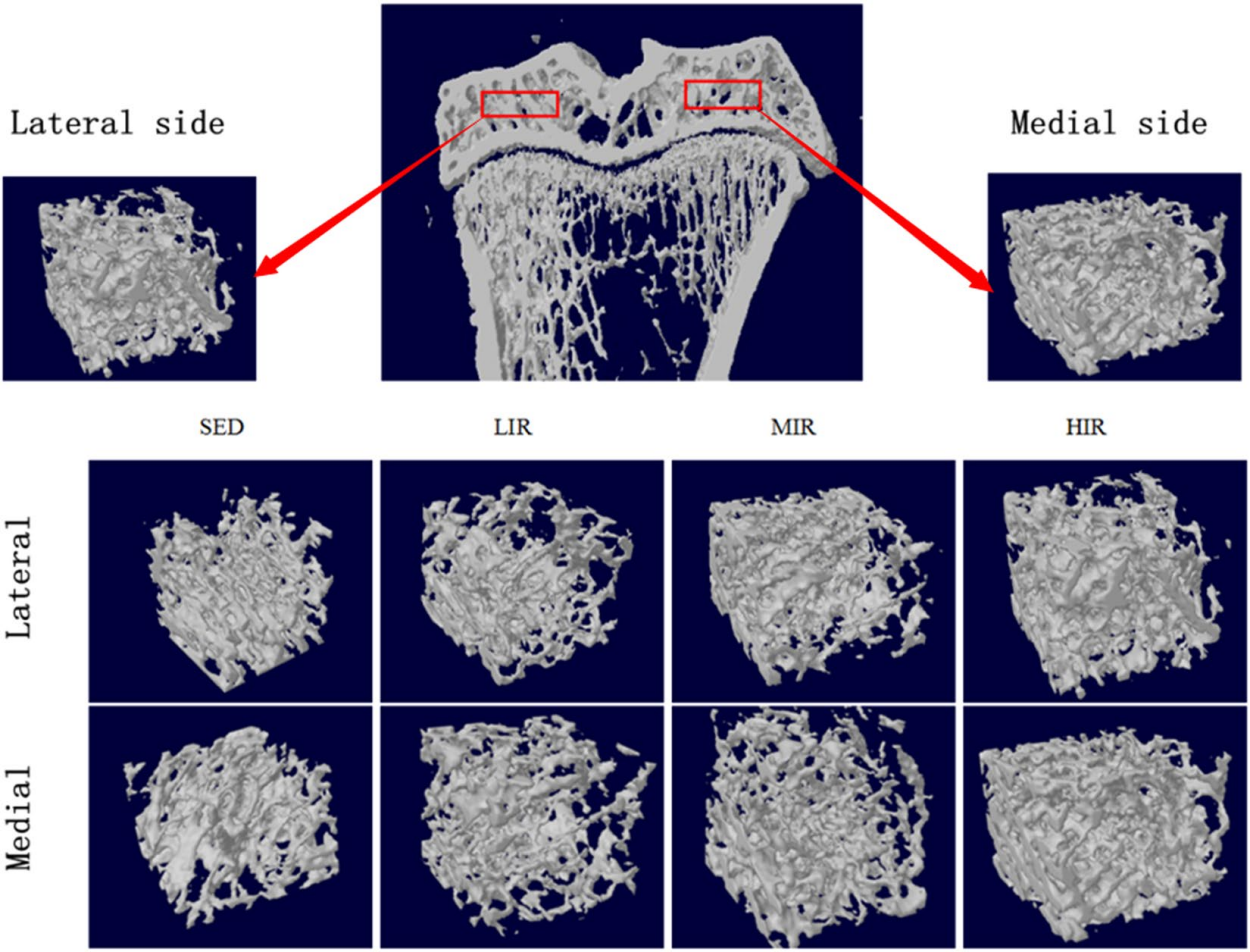
Micro-CT analysis of tibia subchondral trabecular bone showing the ROI selection and diaphragmatic images of both medial and lateral sides in four groups.

The tibia subchondral trabecular bone was also examined by micro-CT scan. Figure 2 presents the ROI selection and diaphragmatic images of both medial and lateral sides in four groups. Results of Micro-CT analysis on 3D microarchitecture parameters of subchondral trabecular bone are summarized in Table 1. Compared with group SED, significant increase in BMD is detected in group HIR on both the lateral (p = 0.035) and medial (p = 0.002) sides. HIR leads to significantly higher bone volume/total volume (BV/TV, %) than controls on the medial side (p = 0.026), indicating a stimulatory effect on trabecular bone formation. In addition, HIR also leads to an increase in trabecular bone thickness on both the medial (p = 0.012) and lateral (p = 0.027) sides, as well as a decrease in trabecular separation on the medial side (p = 0.047). Together with the decreased values of structure model index (SMI), and connectivity density (CD) induced by HIR, our results indicate that HIR led to more and denser trabecular bone with a more plate-like architecture. On the other hand, compared with group SED, there are no obvious changes in either LIR or MIR group, except for an increase in BMD in group MIR on the medial side (p = 0.004), as well as in trabecular number (Tb.N) in group MIR on the lateral side (p = 0.005), and group LIR on the medial side (p = 0.046).

**Table 1:** Quantitative Micro-CT analysis of subchondral trabecular bone (n = 6).

		SED	LIR	MIR	HIR
BMD (g/cm^3^)	Lateral	1.031 ± 0.006	1.039 ± 0.019	1.046 ± 0.013	1.106 ± 0.021*
	Medial	1.040 ± 0.006	1.052 ± 0.021	1.094 ± 0.011*	1.107 ± 0.005*
BV/TV (%)	Lateral	45.77 ± 2.936	45.78 ± 0.519	48.08 ± 0.806	50.98 ± 4.113
	Medial	36.89 ± 2.541	41.22 ± 3.453	44.54 ± 1.501	54.62 ± 3.774*
TbTh (mm)	Lateral	0.141 ± 0.002	0.152 ± 0.012	0.131 ± 0.012	0.176 ± 0.005*
	Medial	0.148 ± 0.002	0.142 ± 0.015	0.129 ± 0.017	0.183 ± 0.002*
Tb.N (1/mm)	Lateral	2.338 ± 0.178	2.541 ± 0.356	2.947 ± 0.174^#^	2.215 ± 0.217
	Medial	2.367 ± 0.348	2.471 ± 0.297*	3.164 ± 0.045	2.943 ± 0.159
Tb.Sp (mm)	Lateral	0.178 ± 0.015	0.177 ± 0.021	0.193 ± 0.018	0.133 ± 0.004
	Medial	0.227 ± 0.044	0.164 ± 0.012	0.195 ± 0.083	0.111 ± 0.007*
SMI	Lateral	1.471 ± 0.092	1.369 ± 0.104	1.487 ± 0.120	0.859 ± 0.092*
	Medial	1.429 ± 0.095	1.212 ± 0.302	1.656 ± 0.073	0.861 ± 0.184*
Conn.Dn	Lateral	63.85 ± 3.658	56.03 ± 1.027	57.09 ± 6.418	45.66 ± 3.797*
	Medial	70.72 ± 3.881	77.29 ± 7.243	69.13 ± 7.771	43.16 ± 2.145*
DA	Lateral	0.785 ± 0.033	0.833 ± 0.043	0.807 ± 0.041	0.736 ± 0.023*
	Medial	0.776 ± 0.035	0.812 ± 0.025	0.833 ± 0.037	0.742 ± 0.025*

*p < 0.05 compared to SED group; ^#^p < 0.05 compared to HIR group.

#### Results from Raman spectra

**Figure 3:**
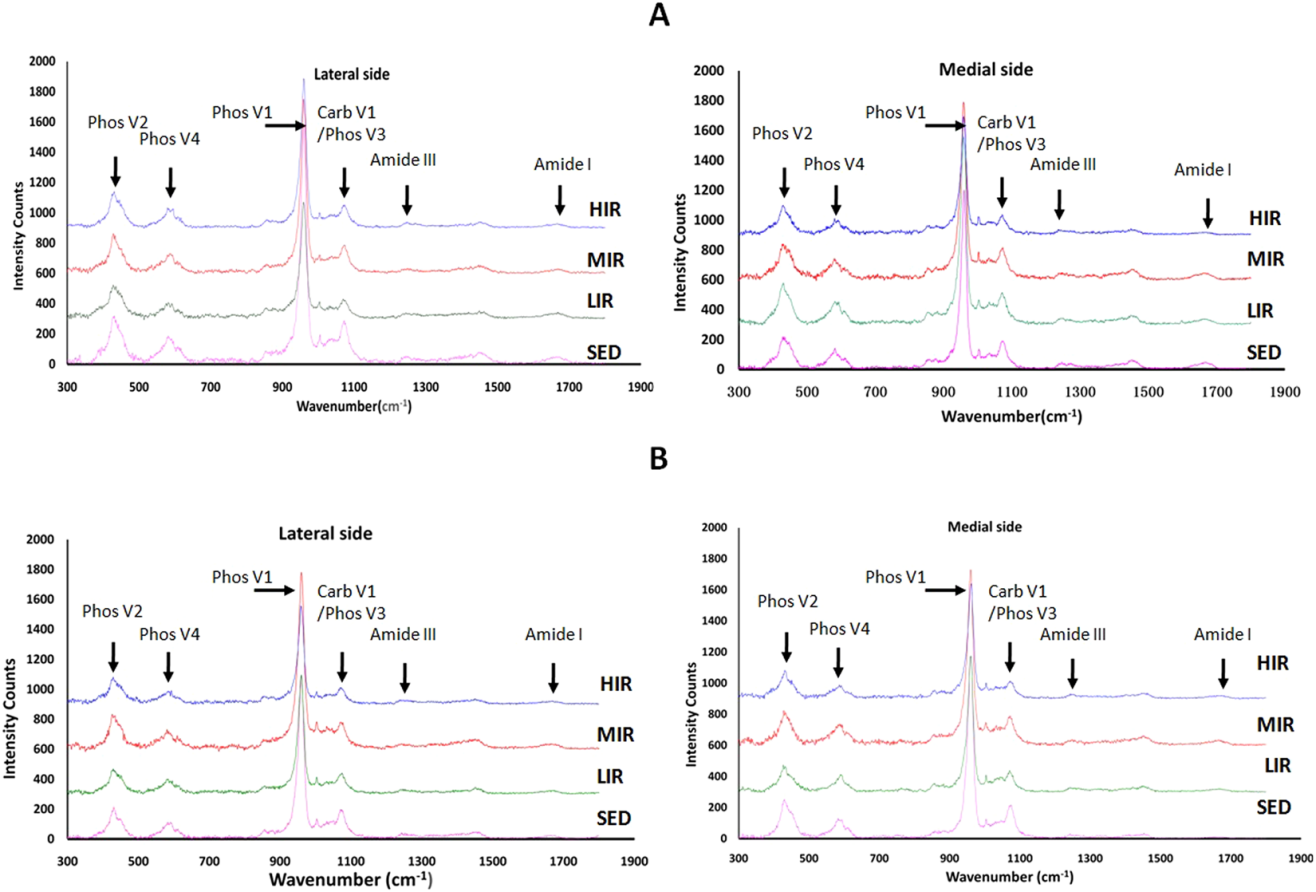
Typical Raman spectra from subchondral plate (**A**) and trabecular bone (**B**) on the lateral and medial side in four groups.

Figure 3A and B illustrates typical Raman spectra from subchondral plate and subchondral trabecular bone on the lateral and medial side in four groups, respectively.

#### Subchondral plate

**Figure 4:**
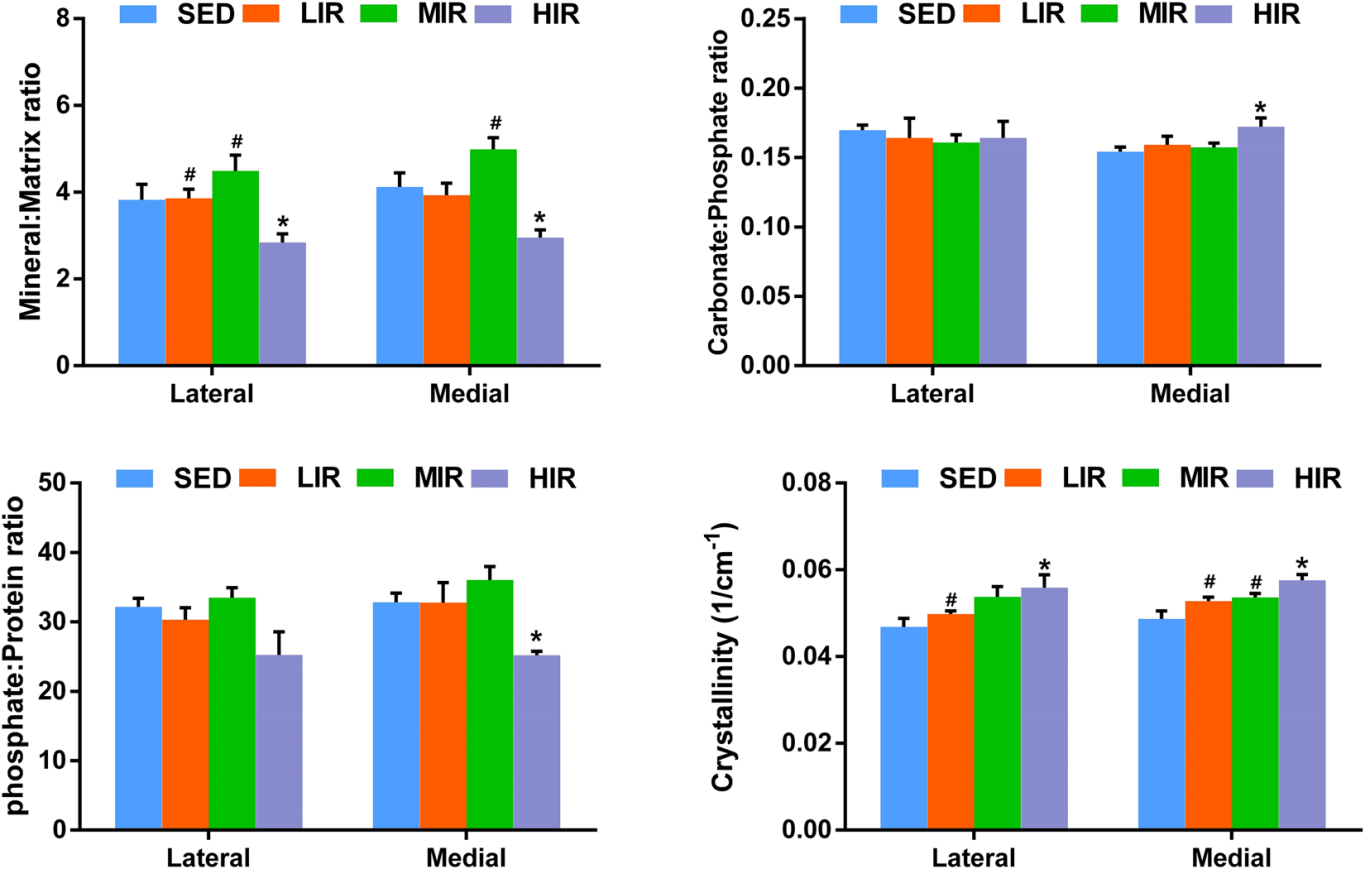
The results of mineral-to-matrix ratio, carbonate-to-phosphate ratio, phosphate-to-protein ratio, and mineral crystallinity of subchondral plate in medial and lateral sites, obtained from Raman spectra. *p < 0.05 compared to SED group at corresponding side. ^#^p < 0.05 compared to HLR group at corresponding side.

Figure 4 describes the results of mineral-to-matrix ratio, carbonate-to-phosphate ratio, phosphate-to-protein ratio, and mineral crystallinity of subchondral plate in four groups. HIR led to a decrease in mineral-matrix ratio on both the medial (p = 0.021) and lateral (p = 0.028) side, indicating a less mineralized subchondral plate following HIR. There was a significantly higher carbonate–phosphate ratio (p = 0.004) and a significantly lower phosphate–protein ratio (p = 0.032) on the medial side in group HIR when compared with group SED, which suggested that HIR resulted in an increased remodeling of subchondral plate. Crystallinity in group HIR was significantly higher than that group SED on the medial (p = 0.002) and lateral side (p = 0.006), respectively. Results from Raman spectra indicate that HIR led to an increased remodeling and less mineralized subchondral plate with increased mineral crystallinity.

#### Subchondral trabecular bone

**Figure 5:**
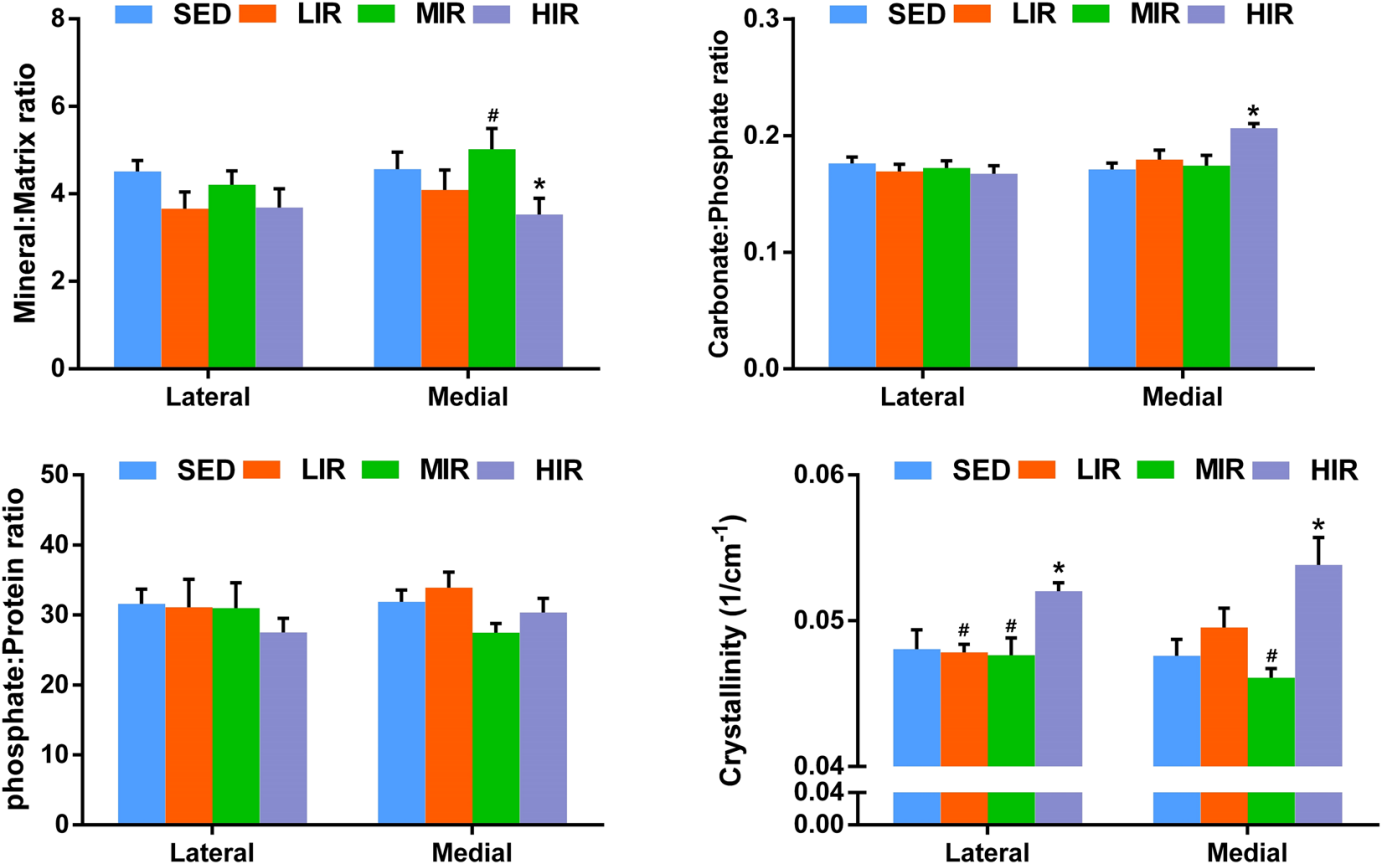
The results of mineral-to-matrix ratio, carbonate-to-phosphate ratio, phosphate-to-protein ratio, and mineral crystallinity of trabecular bone in medial and lateral sites, obtained from Raman spectra. *p < 0.05 compared to SED group at corresponding side. ^#^p < 0.05 compared to HLR group at corresponding side.

Figure 5 shows mineral-to-matrix ratio, carbonate-to-phosphate ratio, phosphate-to-protein ratio, and mineral crystallinity of subchondral trabecular bone in four groups. HIR led to a decrease in mineral-matrix ratio on the medial side (p = 0.033), indicating a less mineralized subchondral trabecular bone following HIR. When compared with group SED, a significantly higher carbonate–phosphate ratio (p = 0.002) was observed on the medial side in group HIR, suggesting that HIR resulted in an increased remodeling of subchondral trabecular bone. Crystallinity in HIR group was significantly increased than that in SED group on both the medial (p = 0.002) and lateral side (p = 0.006). Taken together, similar to subchondral plate, HIR resulted in an increased remodeling and less mineralized subchondral trabecular bone with increased mineral crystallinity.

### Results from microhardness test

#### Subchondral plate

**Figure 6:**
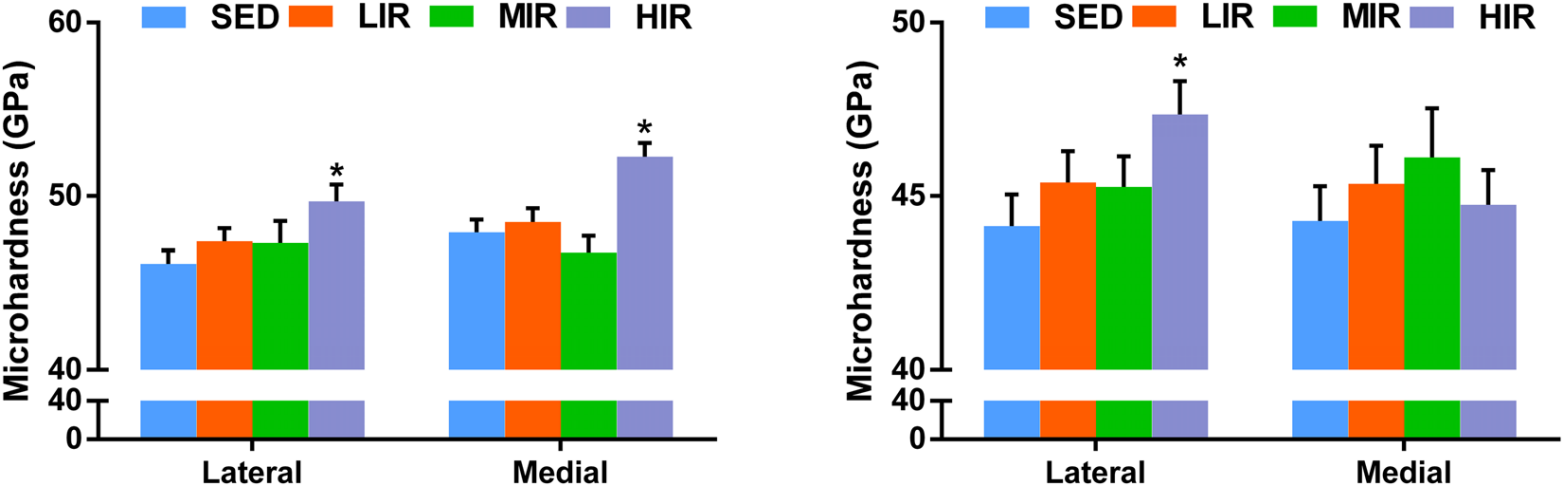
The results of microhardness of subchondral plate (**A**) and subchondral trabecular bone (**B**) in lateral and medial sides in four groups. *p < 0.05 compared to SED group at corresponding side.

Figure 6A shows microhardness of subchondral plate on the lateral and medial sides in four groups. Group HIR exhibits significantly higher microhardness than group SED on the medial (52.27 ± 0.78 GPa vs. 47.92 ± 0.72 GPa, p = 0.001) and lateral side (49.7 ± 0.96 GPa vs. 46.1 ± 0.78 GPa, p = 0.002), respectively. When comparing with group SED, there is no statistical difference in either group LIR or MIR.

#### Subchondral trabecular bone

Figure 6B illustrates microhardness of subchondral trabecular bone on the lateral and medial sides in four groups. Significant higher microhardness in HIR group (48.26 ± 0.95 GPa) was observed on the lateral side when compared with SED group (45. 43 ± 0.91 GPa) (p = 0.031).

## Discussion

Bone remodeling and cartilage maintenance are strongly influenced by biomechanical signals generated by mechanical loading^6^. As one of the most common weight-bearing activity, the effect of running was examined on rat tibial SB in this study, in which the results suggested an intensity-dependent effect on the microarchitecture and composition of SB. In addition, the HIR-induced changes in SB are hypothesized to be contributory factors in the degenerative changes in overlying articular cartilage, demonstrated in our previous study^7^.

Although the effect of running has been extensively examined on bone^8–11^, little is known on SB. After comparing the effect of high intensity and low intensity racing training on equine SB, Murray *et al*. found that high intensity training was associated with increased subchondral bone thickness, increased bone modelling and reduced bone resorption at high load sites^12^. Similar running intensity-related variations were revealed in the present study. In both subchondral plate and trabecular bone, HIR led to highly remodeled, less mineralized, and stiffer SB with considerably altered architecture, while LIR and MIR failed to result in considerable changes in microstructure, composition and hardness. Joint loading was proposed to induce a periodic alteration in the pressure in the medullary cavity^16^. We previously demonstrated that running-induced mechanical stimulus may induce an intensity-dependent effect on the osteogenic and adipogenic differentiation and proliferation of bone marrow stromal cells^17^, which may provide an explanation for the intensity-dependent effect on SB revealed in the present study. On the other hand, bone remodeling and cartilage maintenance are strongly influenced by biomechanical signals generated by mechanical loading^6^. Using the same animal model, our previous study found that LIR and MIR would maintain the integrity of cartilage, while HIR would lead to degenerative changes in articular cartilage^7^. Articular cartilage and its supporting SB are viewed as an authentic functional unit^1, 2^. Thus, our previous and present studies support the concept that a tight interrelationship exists between bone and cartilage in health and disease^6^. Since the effect of running on both cartilage and SB highly depends on its intensity, future investigations are warranted to understand how they communicate under different conditions.

In the present study, HIR-induced changes in SB were characterized in both subchondral plate and trabecular bone. At both sites, considerable changes were identified in ultrastructure and organization, which should be an adaptation to the changes in the biomechanics of the joint, and an attempt to repair micro-damage. Significantly higher BMD was found at subchondral plate and trabecular bone following HIR, which may be harmful for cartilage homeostasis and could predispose to OA initiation^18^. Trabecular bone thickening leads to bone sclerosis, which in turn causes decreased shock absorbency and cartilage damage^19^. Subchondral plate thickening may enhance the molecule crosstalk between articular cartilage and subchondral bone, and further enhance the catabolism of articular cartilage^20^. In addition, change from rod-like to plate-like identified in subchondral trabecular bone of rat submitted to HIR may make subchondral bone stiff^21^ and harmful to articular cartilage. Meanwhile, lower degree of anisotropy (DA) identified in HIR group indicated inferior capability of subchondral trabecular bone to transfer load from cartilage^22^. All these changes are supposed to contribute to the degenerative changes in overlying articular cartilage^7^.

In addition to the changes in ultrastructure and organization, HIR-induced negative effects on the composition and mechanical properties of SB were also reflected by results of Raman spectroscopy and microhardness test. Our findings from Raman spectroscopy indicated that HIR led to decreased bone mineralization, increased remodeling, and increased mineral crystallinity of subchondral plate and trabecular bone. Increased remodeling means that a great amount of the existing bone tissue is relatively young. Because bone tissue continues to mineralize over time, this younger bone tissue contains relatively less mineral than older existing bone tissue, resulting in a decrease in mineral–matrix ratio observed in our study^23^. The highly remodeling and less mineralized SB should be less resistant to the mechanical loading. Additionally, high bone turnover increases the release of various cytokines from SB, which can lead to cartilage degeneration. What is more, with increased mineral crystallinity, the tissue-level strength and stiffness increased while the ductility reduced^24^. In other words, following HIR, SB becomes “brittle” and unable to transfer load for cartilage efficiently, which would further impair the overlying articular cartilage^25^. Meanwhile, the increased stiffness identified by our microhardness test may decrease the viscoelastic properties of SB and produce a loss of its shock absorbing capacity, which in turn causes significant extra mechanical load and subsequent breakdown of the overlying cartilage^19, 26–28^. Taken together, our findings suggested that HIR-induced change in organization and composition of SB may alter its mechanical property, making the SB “brittle and stiff” and adversely affecting the overlying articular cartilage^25^. Nevertheless, further investigations are warranted to back up our findings through analysis of the bone tissue and assessment of the quality of the overlying cartilage with different exercise regimes, using various methods and at different time points.

It is widely recognized that OA is an organ disease that affects the whole joint, where the subchondral bone plays a crucial role. As such, SB is currently recognised as a key target in OA treatment^4^. Since OA is thought to be a multifactorial disease, the changes of SB have been investigated in various animal OA models, including collagenase injection^29^, anterior cruciate ligament transaction (ACLT)^13, 30, 31^, ACLT-menisectomy^32, 33^, menisectomy^34^, monosodium iodoacetate injection^35^, spontaneous OA^15, 36^, groove^13^ and ovariectomy^14^. However, inconsistent results were reported, which are largely due to different types of model used^13^. This suggests that, when searching for treatments targeting on SB, OA patients should be classified into subgroups according to the predominant pathophysiological mechanism involved. Of note, irrespective of the different changes in SB, similar cartilage damage was reported in these models, which highlights the importance of SB for cartilage homeostasis. The increased BMD found in this study fits with previous studies in spontaneous OA model^15, 36^, but contrasts with the study in ACLT-induced model^13, 30, 31^. Previously, Herrero-Beaumont *et al*. proposed that both high and low BMD conditions may be harmful for cartilage homeostasis and could predispose to OA initiation^37^. Meanwhile, the increased bone volume fraction and thickness of SB in this study matches previously reported data in spontaneous OA model^15, 36^, but is opposite to the results in such models as ACLT-^13, 30, 31^, ACLT-meniscectomy-^32, 33^, and groove-induced models^13^. As above mentioned, the increased bone volume fraction and thickness of SB may be harmful to the overlying cartilage. On the other hand, the thinning subchondral bone plate can promote vascular invasion of cartilage and diffusion of molecules from the damaged cartilage, which will improve communication between the biochemical bone and cartilage, and promote the occurrence of OA^38^. The fact that different changes in SB lead to similar cartilage damage probably indicates that, only when the adaptative changes of the underlying SB are stabilized within a certain range can cartilage integrity be maintained. Outside this range, both extreme situations may have deleterious effects on the overlying cartilage.

## Materials and Methods

### Experimental animals, exercise protocols, and sample preparation

A total of 24 male Wistar rats (13–14 weeks old) were randomly and evenly assigned to one of four groups as follows: (1) sedentary control (SED, n = 6); (2) low-intensity running (LIR, n = 6); (3) moderate-intensity running (MIR, n = 6); and (4) high-intensity running (HIR, n = 6). All animals were housed in cages under controlled light/dark cycles (12/12 h) and controlled temperature (22 ± 1 °C), and provided with food and water *ad libitum*. The complete details of the entire study design and procedures involved were in accordance with the Declaration of Helsinki. This study was approved by the animal ethics committee of Nanfang hospital, Southern Medical University.

Animals in group SED maintained a sedentary lifestyle. According to running protocols described previously^7^, those in LIR, MIR, and HIR groups were subjected to treadmill running once a day, 5 days a week for 8 weeks on a motor driven treadmill designed for rodents at a constant speed and inclination which varied according to the following schema: LIR: 15.2 m/min with 0° of inclination for 60 min; MIR: 19.3 m/min with 5° of inclination for 60 min; and HIR: 26.8 m/min with 10° of inclination for 60 min.

**Figure 7:**
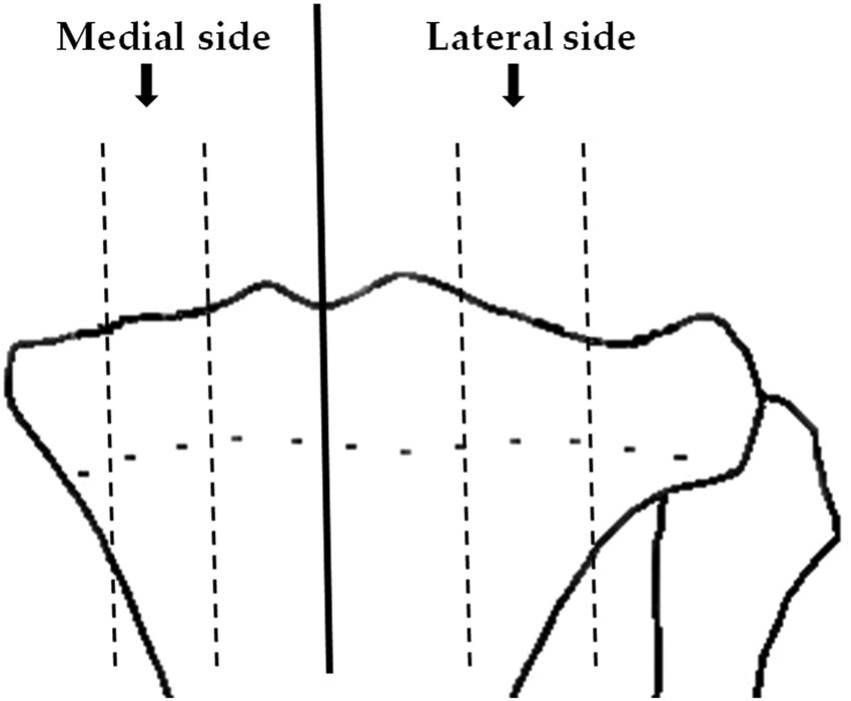
The proximal end of tibia was cut sagittally into lateral and medial side, and for each side, the middle sections were obtained for further examination.

Eight weeks later, all animals were sacrificed under anesthesia by cervical dislocation. Bilateral tibias from each animal were dissected free of soft tissues. The proximal end of the right tibia was used for micro-CT scanning, while the proximal end of the left tibia was collected for further preparation. Briefly, the proximal end of tibia was cut sagittally into lateral and medial side, using a high speed, watercooled saw with a fine diamond coating (EXAKT 300 CP Band System, Norderstedt, Germany). For each side, three sections were obtained by two sagital cuts at 1/3 and 2/3 of its width, respectively (Fig. 7). Afterwards, the middle sections were stored at −80 °C immediately. The sagital surfaces of middle sections were smoothed with polishing paper to increase fineness from 240 grit to 600 grit. The final polishing was carried out on a rotary wheel using 800 grit alumina abrasive in a moist medium. The specimens were sonicated briefly to remove any particles that absorbed on the specimens. Samples were soaked in physiological saline for 12 h under 4 °C before Raman measurements (on one sagital surface) and microhardness testing (on the other surface).

### Micro-CT study

The proximal ends of right tibia were scanned in a micro-CT scanner (SkyScan 1076 Micro-CT system, Kontich, Belgium) with isotropic voxel size of 17.33 µm. The x-ray tube voltage was 88 kV and the current was 100 µA, with a 1.0mm aluminium filter. The scan was carried out through 180 degrees, with a rotation step of 0.6 degrees and an exposure time of 560 ms at each step. The images were reconstructed using NRecon package and then reorientated using Dataviewer to ensure all samples to be analysed in the same orientation.

For analysis of subchondral plate, the load bearing region with an area of 1.04 × 1.04 mm^2^ was selected as ROI (Fig. 1A). BMD, porosity, and thickness of subchondral plate were measured and calculated using CT-analyser software. For analysis of subchondral trabecular bone, a cuboid of trabecular bone with size of 1.04 × 1.04 × 0.52 mm^3^ at beneath the ROI of subchondral plate was selected. BMD (g/cm^3^), BV/TV (%), trabecular thickness (TbTh, mm), trabecular separation (Tb.Sp, mm), trabecular bone pattern factor (TbPf, 1/mm), SMI, and degree of anisotropy (DA) were calculated for subchondral trabecular bone.

### Raman microscopy

Raman spectra were obtained with LabRAM Aramis Raman Spectrometer (Horiba Jobin-Yvon, France). A 785-nm He–Ne laser was focused onto the specimen using a ×50/0.5 NA objective (Olympus, Japan). The radius of the beam was 1.0 μm, and the laser power irradiation over the samples was 17 mW. For each sample, Raman measurement was taken randomly on 4 points from subchondral plate, and subchondral trabecular bone, respectively. For each scan the integration time was 20 s and the final spectrum of each spot was the average of six scans. The Raman system was calibrated with a silicon semiconductor using the Raman peak at 520.7 cm^−1^. All spectra were taken and collected in the region from 300 to 1800 cm^−1^, with a resolution of 0.5 cm^−1^.

Raman spectra of the bone show prominent vibrational bands of organic and inorganic compounds related to bone tissue compositions^39^. However, absolute band intensities in Raman spectroscopy may be affected by Raman scattering efficiency and many other optical effects^20^. Hence, the relative peak intensities of selected pairs of bands were calculated in this study. The mineral to matrix ratio, which indicates the amount of mineralization^40^, was calculated from the intensity of the phosphate v4 (mineral, 580 cm^−1^) peak divided by Amide III (matrix, 1280 cm^−1^) peak in the present study. For remodeling evaluation, the ratios between carbonate-phosphate (1070/960 cm^−1^) and phosphate-protein (960/1660 cm^−1^) were calculated using the Raman band intensities^23^. In addition, the width of the primary phosphate band (near 959 cm^−1^) was measured, and the inverse full-width half-maximal (1/FWHM) was obtained to give an indication of the degree of crystallinity of the mineral part of the bone^41^. The ratios and 1/FWHM were calculated on a pixel-by-pixel basis, and then averaged for each specimen, resulting in a single value for each specimen.

### Microhardness test

Microhardness test is considered a useful method of measurement for bone mechanical properties in small bone specimens. The hardness of a material has been defined as its resistance to penetration by an indenter^42^. Vickers microhardness test was performed using a HMV-2T microhardness test machine fitted with a pyramidal diamond indenter (Shimadzu, Japan). Method was described previously^42^. Briefly, a mass of 40 g was used throughout the test. The pyramidal diamond indenter descent time was set at 10 s interval after which it was allowed to contact the specimen for 15 s. The dimensions of the indentation were measured 45 s after removal of the indenter. The diamond’s diagonal pyramid of indentation was measured microscopically, and the following formula was used to calculate bone hardness. For each bone section, 10 microhardess measurements were taken randomly for subchondral plate and subchondral trabecular bone, respectively.

$${{\rm{H}}}_{{\rm{V}}}=1854.4\times {\rm{L}}/{{\rm{d}}}^{2}$$

where H_V_ stands for Vickers Hardness and is expressed in kg/mm^2^, L is the load in grams and d is the length of the indentation diagonal in mm.

### Statistical analysis

Results are expressed as the mean ± standard error of mean (SEM). Statistical analysis was carried out using SPSS 19.0 analysis software (SPSS Inc., Chicago, IL, USA). Since the sample size is small (n = 6 per group), non-parametric statistical analysis was performed for comparison between each groups. For comparison of the results among medial and lateral sides of each groups, post-hoc multiple comparisons between groups were made using the Kruskal-Wallis test. When significant main effects were found, Wilcoxon rank sum tests were performed for comparison between each groups. A p value < 0.05 was considered to be significant.

## References

[1] Imhof H (2000). Subchondral bone and cartilage disease: a rediscovered functional unit. Invest Radiol.

[2] Lories RJ, Luyten FP (2011). The bone-cartilage unit in osteoarthritis. Nat Rev Rheumatol.

[3] Burr D (2004). Anatomy and physiology of the mineralized tissues: role in the pathogenesis of osteoarthrosis. Osteoarthritis Cartilage.

[4] Castañeda S, Roman-Blas JA, Largo R, Herrero-Beaumont G (2012). Subchondral bone as a key target for osteoarthritis treatment. Biochem Pharmacol.

[5] Kwan TS, Lajeunesse D, Pelletier JP, Martel-Pelletier J (2010). Targeting subchondral bone for treating osteoarthritis: what is the evidence?. Best Pract Res Clin Rheumatol.

[6] Yokota H, Leong DJ, Sun HB (2011). Mechanical loading: bone remodeling and cartilage maintenance. Curr Osteoporos Rep.

[7] Ni GX (2013). Intensity-dependent effect of treadmill running on knee articular cartilage in a rat model. Biomed Res Int.

[8] Iwamoto J, Takeda T, Sato Y (2005). Effect of treadmill exercise on bone mass in female rats. Exp Anim.

[9] Bourrin S, Genty C, Palle S, Gharib C, Alexandre C (1994). Adverse effects of strenuous exercise: a densitometric and histomorphometric study in the rat. J Appl Physiol.

[10] Hind K, Truscott JG, Evans JA (2006). Low lumbar spine bone mineral density in both male and female endurance runners. Bone.

[11] Lappe J (2008). Calcium and vitamin D supplementation decreases incidence of stress fractures in female navy recruits. J Bone Miner Res.

[12] Murray RC, Vedi S, Birch HL, Lakhani KH, Goodship AE (2001). Subchondral bone thickness, hardness and remodelling are influenced by short-term exercise in a site-specific manner. J Orthop Res.

[13] Sniekers YH (2008). A role for subchondral bone changes in the process of osteoarthritis; a micro-CT study of two canine models. BMC Musculoskelet Disord.

[14] Sniekers YH, Weinans H, van Osch GJ, van Leeuwen JP (2010). Oestrogen is important for maintenance of cartilage and subchondral bone in a murine model of knee osteoarthritis. Arthritis Res Ther.

[15] Wang T, Wen CY, Han CH, Lu WW, Chiu KY (2013). Spatial and temporal changes of subchondral bone proceed to microscopic articular cartilage degeneration in guinea pigs with spontaneous osteoarthritis. Osteoarthritis Cartilage.

[16] Zhang P, Su M, Liu Y, Hsu A, Yokota H (2007). Knee loading dynamically alters intramedullary pressure in mouse femora. Bone.

[17] Liu, S. *et al*. Intensity-dependent effect of treadmill running on differentiation of rat bone marrow stromal cells. Molecular Medicine Reports. In Press. 10.3892/mmr.2018.8797 PMC5983966 29620179

[18] Ding M, Odgaard A, Hvid I (2003). Changes in the three-dimensional microstructure of human tibial cancellous bone in early osteoarthritis. J Bone Joint Surg Br.

[19] Radin EL, Rose RM (1986). Role of subchondral bone in the initiation and progression of cartilage damage. Clin Orthop.

[20] Nakamura T, Mizuno S (2010). The discovery of hepatocyte growth factor (HGF) and its significance for cell biology, life sciences and clinical medicine. Proc Jpn Acad Ser B Phys Biol Sci.

[21] Ding M, Odgaard A, Danielsen CC, Hvid I (2002). Mutual associations among microstructural, physical and mechanical properties of human cancellous bone. J Bone Joint Surg Br.

[22] Goulet RW (1994). The relationship between the structural and orthogonal compressive properties of trabecular bone. J Biomech.

[23] McCreadie BR (2006). Bone tissue compositional differences in women with and without osteoporotic fracture. Bone.

[24] Yerramshetty JS, Akkus O (2008). The associations between mineral crystallinity and the mechanical properties of human cortical bone. Bone.

[25] Day JS (2001). A decreased subchondral trabecular bone tissue elastic modulus is associated with pre-arthritic cartilage damage. J Orthop Res.

[26] Radin EL (1991). Mechanical determinants of osteoarthrosis. Semin Arthritis Rheum.

[27] Radin EL (1984). Effects of mechanical loading of the tissues of the rabbit knee. J Orthop Res.

[28] Shimizu M, Tsuji H, Matsui H, Ratoh Y, Sano A (1993). Morphometric analysis of subchondral bone of the tibial condyle in osteoarthrosis. Clin Orthop.

[29] Botter SM (2011). Osteoarthritis induction leads to early and temporal subchondral plate porosity in the tibial plateau of mice: an *in vivo* microfocal computed tomography study. Arthritis Rheum.

[30] Boyd SK, Muller R, Leonard T, Herzog W (2005). Long-term periarticular bone adaptation in a feline knee injury model for posttraumatic experimental osteoarthritis. Osteoarthritis Cartilage.

[31] Hayami T (2004). The role of subchondral bone remodeling in osteoarthritis: reduction of cartilage degeneration and prevention of osteophyte formation by alendronate in the rat anterior cruciate ligament transection model. Arthritis Rheum.

[32] McErlain DD (2008). Study of subchondral bone adaptations in a rodent surgical model of OA using *in vivo* micro-computed tomography. Osteoarthritis Cartilage.

[33] Intema F (2010). In early OA, thinning of the subchondral plate is directly related to cartilage damage: results from a canine ACLT-meniscectomy model. Osteoarthritis Cartilage.

[34] Fahlgren A, Messner K, Aspenberg P (2003). Meniscectomy leads to an early increase in subchondral bone plate thickness in the rabbit knee. Acta Orthop Scand.

[35] Morenko BJ (2004). *In vivo* micro computed tomography of subchondral bone in the rat after intra-articular administration of monosodium iodoacetate. Contemp Top Lab Anim Sci.

[36] Layton MW (1988). Examination of subchondral bone architecture in experimental osteoarthritis by microscopic computed axial tomography. Arthritis Rheum.

[37] Herrero-Beaumont G, Roman-Blas JA, Largo R, Berenbaum F, Castaneda S (2011). Bone mineral density and joint cartilage: four clinical settings of a complex relationship in osteoarthritis. Ann Rheum Dis.

[38] Buckwalter JA, Mankin HJ (1998). Articular cartilage: degeneration and osteoarthrosis, repair, regeneration, and transplantation. Instr Course Lect.

[39] Morris MD, Mandair GS (2011). Raman Assessment of Bone Quality. Clin Orthop.

[40] Wopenka B, Kent A, Pasteris JD, Yoon Y, Thomopoulos S (2008). The tendon-to-bone transition of the rotator cuff: a preliminary Raman spectroscopic study documenting the gradual mineralization across the insertion in rat tissue samples. Appl Spectrosc.

[41] Matousek P (2006). Noninvasive Raman spectroscopy of human tissue *in vivo*. Appl Spectrosc.

[42] Ni GX (2007). Mechanical properties of femoral cortical bone following cemented hip replacement. J Orthop Res.

